# RASSF1 is identified by transcriptome coordination analysis as a target of ATF4

**DOI:** 10.1002/2211-5463.13569

**Published:** 2023-02-14

**Authors:** Youwen Zhang, Kim‐Tuyen Huynh‐Dam, Xiaokai Ding, Vitali Sikirzhytski, Chang‐uk Lim, Eugenia Broude, Hippokratis Kiaris

**Affiliations:** ^1^ Department of Drug Discovery and Biomedical Sciences, College of Pharmacy University of South Carolina Columbia SC USA; ^2^ Peromyscus Genetic Stock Center University of South Carolina Columbia SC USA

**Keywords:** ATF4, ER stress, RASSF1, transcriptome coordination analysis, unfolded protein response

## Abstract

Evaluation of gene co‐regulation is a powerful approach for revealing regulatory associations between genes and predicting biological function, especially in genetically diverse samples. Here, we applied this strategy to identify transcripts that are co‐regulated with unfolded protein response (UPR) genes in cultured fibroblasts from outbred deer mice. Our analyses showed that the transcriptome associated with *RASSF1*, a tumor suppressor involved in cell cycle regulation and not previously linked to UPR, is highly correlated with the transcriptome of several UPR‐related genes, such as *BiP*/*GRP78*, *DNAJB9*, *GRP94*, *ATF4*, *DNAJC3*, and *CHOP*/*DDIT3*. Conversely, gene ontology analyses for genes co‐regulated with *RASSF1* predicted a previously unreported involvement in UPR‐associated apoptosis. Bioinformatic analyses indicated the presence of ATF4‐binding sites in the *RASSF1* promoter, which were shown to be operational using chromatin immunoprecipitation. Reporter assays revealed that the *RASSF1* promoter is responsive to ATF4, while ablation of *RASSF1* mitigated the expression of the ATF4 effector *BBC3* and abrogated tunicamycin‐induced apoptosis. Collectively, these results implicate RASSF1 in the regulation of endoplasmic reticulum stress‐associated apoptosis downstream of ATF4. They also illustrate the power of gene coordination analysis in predicting biological functions and revealing regulatory associations between genes.

## Graphical abstract

*RASSF1* is a tumor suppressor involved in cell cycle regulation and not previously linked to unfolded protein response (UPR). By evaluating gene coordination in genetically diverse samples, we found *RASSF1* is highly correlated with transcriptomes of several UPR‐related genes. Our further investigations identified *RASSF1* as a target of ATF4 in the regulation of endoplasmic reticulum (ER) stress‐associated apoptosis.

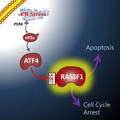

**Abbreviations**
ATF4: activating transcription factor 4
BBC3: B‐cell lymphoma (BCL)‐2‐binding component 3
BH3: B‐cell lymphoma (BCL)‐2 homology domain 3
BiP: binding immunoglobulin protein
CARE: C/ebp‐Atf response element
CCNA2: cyclin A2
CHOP: C/EBP homologous protein
DAXX: death domain‐associated protein
DDIT3: DNA damage‐inducible transcript 3
DNAJB9: DnaJ homolog subfamily B member 9
DNAJC3: DnaJ homolog subfamily C member 3
eIF2α: eukaryotic initiation factor 2α
ER: endoplasmic reticulum
GADD45A: growth arrest and DNA damage‐inducible protein 45 alpha
GAPDH: glyceraldehyde‐3‐phosphate dehydrogenase
GRP78: glucose‐regulating protein 78
HAUSP: herpesvirus‐associated ubiquitin‐specific protease
HSP90B1: heat shock protein 90 beta family member 1
HSPA5: heat shock protein family A member 5
IRE1: inositol‐requiring enzyme 1
ISRIB: integrated stress response inhibitor
MDM2: murine double minute 2
PERK: protein kinase R (PKR)‐like endoplasmic reticulum kinase
PUMA: p53 upregulated modulator of apoptosis
RASSF1: Ras association domain family member 1
RCAN1: regulator of calcineurin 1
UPR: unfolded protein response

Differential analysis of gene expression is a powerful and extensively used strategy for pointing to regulatory relationships between genes [1, 2]. Nevertheless, its applicability is highly limited when genetically diverse specimens are analyzed because they result in high variation in gene expression. Thus, highly variable, albeit biologically significant transcripts are overlooked because they do not pass the stringency thresholds of differential expression. Conversely, even subtle changes in expression levels may point to transcripts with minimal involvement in specific processes when variation is narrow [3, 4, 5].

To overcome the limitations of conventional differential expression analysis we focused on the analysis of patterns of gene co‐expression in genetically diverse specimens. By concentrating on the co‐regulation of genes associated with the unfolded protein response (UPR) in specimens from outbred deer mice (*Peromyscus*), we showed that despite the variation in the levels of expression of individual genes, a striking correlation is maintained in their levels in samples from different individuals [6]. This correlation extends to the correlation of the UPR genes with the whole transcriptome and exhibits different profiles when endoplasmic reticulum (ER) stress is induced and pathology is inflicted [7, 8, 9]. Beyond the UPR, this approach was also shown to be especially meaningful when the pattern of gene coordination was evaluated, at the whole transcriptome level, in outbred genetically diverse specimens. For example, in people suffering from frailty syndrome, this approach readily manifested the involvement of the immune system [10]. In brain samples of different species of deer mice, it pointed to a loss of smell at aging and identified transcriptomic coordination differences that accompany the development of histological changes consistent with neurodegeneration [11]. In analyses of liver samples from deer mice receiving a high‐fat diet, this strategy demonstrated the engagement of the immune system, prior to the development of histologically detectable inflammation [12].

In the present study, we sought to exploit this analysis towards the discovery of specific transcripts that may play unrecognized roles as yet in specific processes. We specifically hypothesized that in genetically diverse specimens, transcripts with causal involvement in certain biochemical pathways should exhibit high coordination, with various genes known to be involved in these pathways. Furthermore, it is plausible that this coordination extends beyond the expression of individual genes, to the whole transcriptome, and can be reflected to how tightly each and every gene is co‐expressed between the interrogated transcript and known gene targets of the pathway in question.

The UPR was selected for this analysis because it represents a central homeostatic response at which different biochemical pathways converge during the stress of the ER [13, 14]. Furthermore, it is associated with considerable changes in gene expression profiles that vary among individuals [6, 15, 16]. Our analyses pointed to *RASSF1* that exhibited high coordination with multiple UPR genes. *RASSF1* is a tumor suppressor that has an established role in cell cycle regulation and apoptosis but no links to UPR previously [17, 18, 19]. A combination of *in silico* predictions that were based on coordination studies and gene ontology analyses, combined with validation experiments *in vitro*, identified *RASSF1* as a UPR target, operating in a manner according to which during ER stress the UPR‐related transcription factor ATF4 activates *RASSF1* transcription by interacting directly with its promoter. In turn, RASSF1 induces cell cycle arrest and apoptosis. The results, besides implicating causally the response of *RASSF1* to ER stress, also illustrate how gene coordination analysis can be applied to genetically diverse specimens and reveal novel associations between genes and specific biological processes.

## Results

### Whole transcriptome coordination between *RASSF1* and UPR target genes

**Fig. 1:**
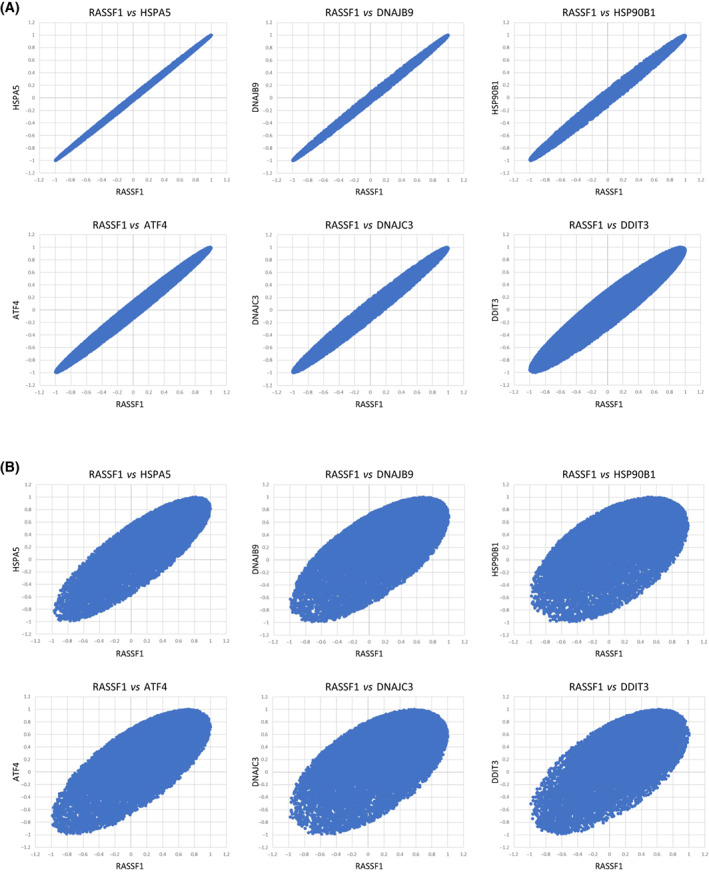
Whole transcriptome coordination analysis shows positive correlations between *RASSF1* and UPR target genes. (A) Scatterplots showing the *R* (Pearson's) values for the whole transcriptomes of *RASSF1* and each of UPR target genes *HSPA5/BiP*, *DNAJB9*, *HSP90B1*, *ATF4*, *DNAJC3*, and *DDIT3* in primary fibroblasts of deer mice with or without tunicamycin treatment (*n* = 6). The data are shown in Table S1, and the methods can be found in our previous publication [8]. (B) Scatterplots showing the *R* (Pearson's) values for the whole transcriptomes of *RASSF1* and each of UPR target genes *HSPA5/BiP*, *DNAJB9*, *HSP90B1*, *ATF4*, *DNAJC3*, and *DDIT3* in human liver specimens (*n* = 6). The data are shown in Table S2, and the methods can be found in our previous publication [7].

Earlier observations showed that UPR‐associated genes exhibit coordinated expression, not only between their individual expression levels but also when the correlation of each with the whole transcriptome was evaluated and compared to that of other UPR genes, in pairwise comparisons [8]. Thus, we hypothesized that genes that have causative involvement in the UPR will also show highly coordinated expression, at the whole transcriptome level, with that of established UPR target genes. To test this hypothesis, we initially calculated the Pearson's correlation coefficient of the expression of a panel of UPR genes with the whole transcriptome (Table S1). The analysis was performed in primary fibroblasts isolated from different, outbred deer mouse individuals, which were cultured in the presence or absence of tunicamycin. The gene that exhibited the highest correlation with *BiP/GRP78/HSPA5*, the major UPR regulator [20, 21], was *RASSF1* that also exhibited high correlation with various UPR targets as well (Fig. 1A). To explore whether the coordination identified is conserved across experimental and biological systems, we also performed the same analysis in RNA‐Seq data of human liver specimens [7] (Table S2) and found similar relationships between *RASSF1* and UPR target genes although the correlations are not as tight as those in primary fibroblasts (Fig. 1B). *RASSF1* (Ras association domain‐containing protein 1) encodes for a Ras effector protein that has been studied primarily in the context of tumorigenesis [22, 23]. It is a tumor suppressor gene and its expression is lost in human cancers by mechanisms that usually involve aberrant DNA methylation. No evidence to our knowledge exists linking *RASSF1* with the UPR yet. As shown in Fig. 1, an astonishing degree of coordination was unveiled with all UPR targets examined, implying a potential role of RASSF1 in the regulation of the UPR. Interestingly, the association was only reduced with *CHOP* (wider plot in Fig. 1A, lower right, as compared to other combinations), which is consistent with the fact that *CHOP* is also regulated by alternative to UPR mechanisms [24].

### *In silico* analysis of RASSF1 function

In order to further explore the function of RASSF1, we calculated Pearson's correlation between *RASSF1* and the whole transcriptome and subjected the top 1129 genes (*P* < 0.05 Pearson's) to Gene Ontology analysis for biological function prediction. As shown in Table 1 and Table S3, this analysis showed a striking association with processes relevant to ER stress response, especially in relation to PERK signaling that represents one of the three major branches of the UPR, along with IRE1 and ATF6 [25, 26]. In conformity with these discoveries, coordination analysis showed a tight association between the transcriptomes of *RASSF1* and both *BBC3* and *GADD45A*, two pro‐apoptotic genes primarily induced through the PERK‐eIF2α branch of the UPR [27, 28, 29, 30] while the correlation between the transcriptomes of *RASSF1* and *RCAN1* was less tight, aligning with the fact that *RCAN1* is an ATF6‐dependent, pro‐survival regulator during ER stress [31, 32, 33] (Fig. S1). Other biological processes predicted by this analysis were related to signal transduction and are consistent with known functions of RASSF1 (Table 1).

**Table 1:** Gene ontology enrichment analysis for transcripts that exhibited positively correlated expression (*P* < 0.05, Pearson's) with *RASSF1*.

GO term	Description	*P*‐value	FDR *q*‐value
GO:0034976	Response to endoplasmic reticulum stress	1.03E‐06	6.40E‐03
GO:2001233	Regulation of apoptotic signaling pathway	1.47E‐05	4.58E‐02
GO:1901565	Organonitrogen compound catabolic process	3.89E‐05	8.07E‐02
GO:0033554	Cellular response to stress	5.57E‐05	8.68E‐02
GO:2001235	Positive regulation of apoptotic signaling pathway	8.40E‐05	1.05E‐01
GO:0006986	Response to unfolded protein	1.08E‐04	1.12E‐01
GO:0035966	Response to topologically incorrect protein	1.08E‐04	9.61E‐02
GO:0006915	Apoptotic process	1.22E‐04	9.53E‐02
GO:0030163	Protein catabolic process	1.26E‐04	8.73E‐02
GO:0012501	Programmed cell death	1.77E‐04	1.10E‐01
GO:0060548	Negative regulation of cell death	1.77E‐04	1.00E‐01
GO:1903912	Negative regulation of endoplasmic reticulum stress‐induced eif2 alpha phosphorylation	2.19E‐04	1.14E‐01
GO:0008219	Cell death	3.09E‐04	1.48E‐01
GO:0051246	Regulation of protein metabolic process	4.32E‐04	1.92E‐01
GO:0032270	Positive regulation of cellular protein metabolic process	5.01E‐04	2.08E‐01

### Regulation of *RASSF1* by ATF4

**Fig. 2:**
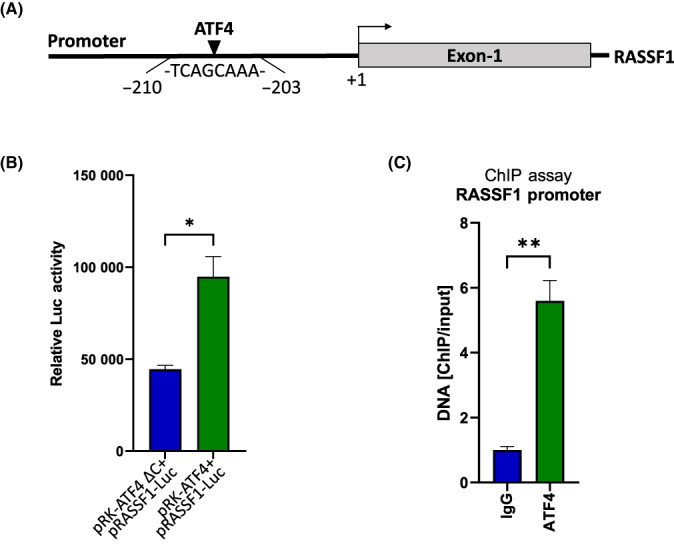
ATF4 occupies the *RASSF1* promoter and regulates its expression. (A) Schema of the *RASSF1* promoter with the localization of putative ATF4‐binding site. (B) Luciferase activity in HEK293FT cells co‐transfected with pRASSF1‐Luc and pRK‐ATF4 or pRK‐ATF4 ΔC (1–275) (*n* = 2 biological replicates). The results were expressed as relative luciferase activity normalized with the total protein concentration. *P*‐value was calculated with an unpaired two‐tailed *t*‐test. (C) Soluble chromatin from HEK293FT cells was precipitated with anti‐ATF4 antibody or rabbit IgG (*n* = 3 biological replicates). The final DNA samples were amplified by qPCR with primers for the *RASSF1* promoter listed in Table 2. The results were expressed as the percentage to the input DNA. *P*‐value was calculated with an unpaired two‐tailed *t*‐test. Data represent the mean ± SEM. *P < 0.05, **P < 0.01.

The fact that our results, so far, were based on RNA expression data, in combination with the prediction that *RASSF1* is associated with the response to ER stress, prompted us to test whether *RASSF1* harbors consensus ER stress‐responsive elements within its promoter. Thus, a ~ 1 kb region in the 5′‐UTR of *RASSF1* was identified and subjected to bioinformatic analysis for the prediction of transcription factor‐binding sites. This analysis readily identified an ATF4‐binding site that was located between −210 and −203 positions from the transcription start site (TSS) of *RASSF1* (Fig. 2A) and the sequence 5′‐TCAGCAAA‐3′ was similar to canonical CARE sequence 5′‐TGATGxAAx‐3′ [34]. ATF4 is an established UPR target downstream of PERK [35, 36].

Subsequently, we tested whether the promoter of *RASSF1* is responsive to ER stress. Thus, a luciferase‐based reporter construct was constructed bearing the *RASSF1* promoter (−931 to +38), and its activity was evaluated following co‐transfection of human embryonic kidney 293FT (HEK293FT) cells with human wild‐type or mutant ATF4 expression plasmids. As shown in Fig. 2B, the activation of luciferase activity in *RASSF1* promoter reporter was significantly higher by the wild‐type ATF4. Chromatin immunoprecipitation studies were also performed and confirmed that ATF4 physically interacts with *RASSF1* promoter (Fig. 2C).

### Induction of *RASSF1* by tunicamycin (Tun) and thapsigargin (Thap)

**Fig. 3:**
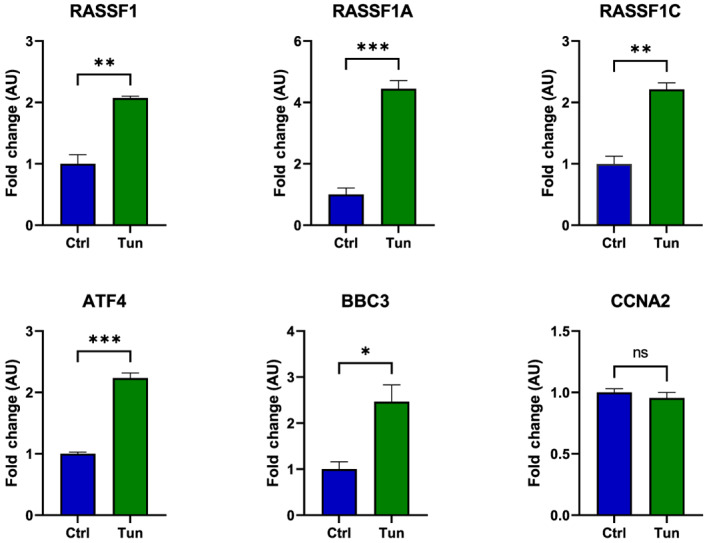
Endoplasmic reticulum stress induced by tunicamycin (Tun) in HEK293FT cells upregulates *RASSF1*, *RASSF1A*, *RASSF1C*, *ATF4*, and *BBC3* expression. HEK293FT cells were treated with tunicamycin (5 μg·mL^−1^), and the relative gene expression was detected by RT‐qPCR using primers listed in Table 2 and normalized with *GAPDH* expression (*n* = 3 biological replicates). Ctrl, control; Tun, tunicamycin treatment. *P*‐values were calculated with an unpaired two‐tailed *t*‐test. Data represent the mean ± SEM. *P < 0.05, **P < 0.01, ***P < 0.001; ns, nonsignificant.

**Fig. 4:**
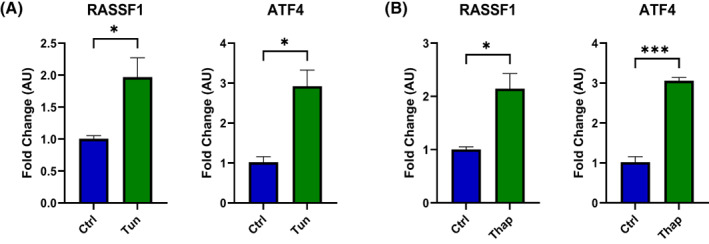
Endoplasmic reticulum stress induced by (A) tunicamycin (Tun) or (B) thapsigargin (Thap) in HFFF2 cells upregulates *RASSF1* and *ATF4* expression. HFFF2 cells were treated with tunicamycin (5 μg·mL^−1^) or thapsigargin (3 μm), and the relative gene expression was detected by RT‐qPCR using primers listed in Table 2 and normalized with *GAPDH* expression (*n* = 3 biological replicates). Tun, tunicamycin treatment; Thap, thapsigargin treatment; Ctrl, control. *P*‐values were calculated with an unpaired two‐tailed t‐test. Data represent the mean ± SEM. *P < 0.05, ***P < 0.001.

**Fig. 5:**
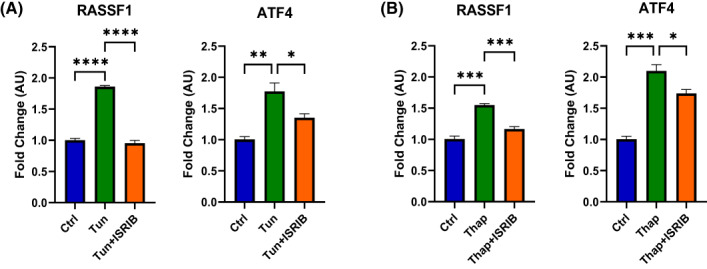
Endoplasmic reticulum stress induced by (A) tunicamycin (Tun) or (B) thapsigargin (Thap) in HEK293FT cells upregulates *RASSF1* and *ATF4* expression, and the effects were reduced by ISRIB addition. HEK293FT cells were treated by tunicamycin (5 μg·mL^−1^) or thapsigargin (3 μm) with or without ISRIB (500 nm) addition, and the relative gene expression was detected by RT‐qPCR using primers listed in Table 2 and normalized with *GAPDH* expression (*n* = 3 biological replicates). Ctrl, control; Thap, thapsigargin treatment; Tun, tunicamycin treatment. *P*‐values were calculated with a one‐way ANOVA followed by the Tukey's multiple comparison test. Data represent the mean ± SEM. *P < 0.05, **P < 0.01, ***P < 0.001, ****P < 0.0001.

The aforementioned results predict that *RASSF1* is a UPR target gene. To test this hypothesis, we exposed HEK293FT and human fetal foreskin fibroblasts (HFFF2) to tunicamycin and thapsigargin, the established UPR activators [37], and monitored the levels of *RASSF1*. As shown in Fig. 3, the levels of both *RASSF1* and of its splice variants *RASSF1A* and *RASSF1C* increased significantly in the tunicamycin‐treated cells, among which the level of *RASSF1A* expression increased more than 4‐fold compared with about 2‐fold increase in *RASSF1C*. Similarly, *ATF4* levels and its downstream target *BBC3* [38, 39] were induced by tunicamycin. The significant induction of *RASSF1* and *ATF4* was also seen in HFFF2 treated with either tunicamycin (Fig. 4A) or thapsigargin (Fig. 4B). In addition, the integrated stress response inhibitor (ISRIB), an established inhibitor of the PERK branch of UPR, significantly reduced the induction of *RASSF1* and *ATF4* in HEK293FT by either tunicamycin (Fig. 5A) or thapsigargin (Fig. 5B).

**Fig. 6:**
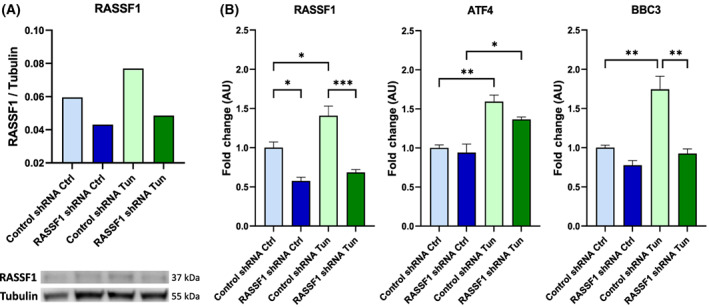
RASSF1 knockdown by shRNA modulates *RASSF1* and its related genes expression in cells under ER stress conditions. (A) The relative expression of RASSF1 protein in HEK293FT cells transfected with hRASSF1 or control shRNA and treated with tunicamycin (5 μg·mL^−1^), detected with western blotting and normalized with α‐Tubulin levels. (B) The relative expressions of *RASSF1*, *ATF4* and *BBC3* mRNA in HEK293FT cells transfected with RASSF1 or control shRNA and treated with tunicamycin, detected with RT‐qPCR and normalized with *GAPDH* expression (*n* = 3 biological replicates). Control shRNA Ctrl—cells transfected with scrambled shRNA and without tunicamycin treatment, RASSF1 shRNA Ctrl—cells transfected with hRASSF1‐shRNA and without tunicamycin treatment, Control shRNA Tun—cells transfected with scrambled shRNA and treated with tunicamycin, and RASSF1 shRNA Tun—cells transfected with hRASSF1‐shRNA and treated with tunicamycin. *P*‐values were calculated with a one‐way ANOVA followed by the Tukey's multiple comparison test. Data represent the mean ± SEM. *P < 0.05, **P < 0.01, ***P < 0.001.

When, however, the expression of *RASSF1* was inhibited by shRNA (Fig. 6A), the tunicamycin‐induced activation of *BBC3*, but not of *ATF4*, was abrogated (Fig. 6B). This is consistent with the notion that RASSF1 is downstream of ATF4 but upstream of BBC3, during tunicamycin‐induced ER stress. Consistent with these findings coordination analysis between *RASSF1* and each of *BBC3* or *CCNA2*‐associated transcriptomes showed coordination with the former but not with the latter (Fig. S1).

### RASSF1‐induced cell cycle arrest and apoptosis during ER stress

**Fig. 7:**
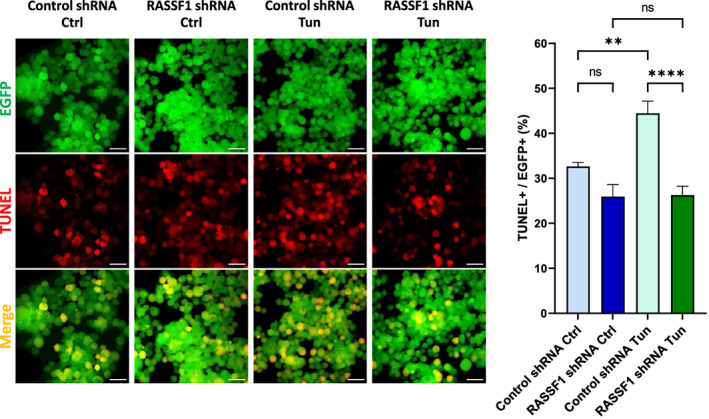
RASSF1 knockdown by shRNA reduces the cell apoptosis under ER stress conditions. HEK293FT cells were transfected with hRASSF1 or control shRNA and treated with tunicamycin (5 μg·mL^−1^). The ratios of TUNEL‐positive to EGFP‐positive cells were detected with the *In Situ* Cell Death Detection Kit and analyzed under a fluorescence microscope (representative images of *n* = 10). Scale bars: 30 μm. Control shRNA Ctrl—cells transfected with scrambled shRNA and without tunicamycin treatment, RASSF1 shRNA Ctrl—cells transfected with hRASSF1‐shRNA and without tunicamycin treatment, Control shRNA Tun—cells transfected with scrambled shRNA and treated with tunicamycin, and RASSF1 shRNA Tun—cells transfected with hRASSF1‐shRNA and treated with tunicamycin. *P*‐values were calculated with a one‐way ANOVA followed by the Tukey's multiple comparison test. Data represent the mean ± SEM. **P < 0.01, ****P < 0.0001; ns, nonsignificant.

In order to functionally evaluate the integration of RASSF1 into UPR signaling, we evaluated the consequences of RASSF1 inhibition in tunicamycin‐induced cell cycle arrest. As shown in Fig. S2, exposure of HEK293FT cells to tunicamycin‐induced G1 cell cycle arrest and reduced the fraction of cells in G2/M phase of cell cycle. However, shRNA‐mediated RASSF1 inhibition reduced the fraction of cells in G1 and increased the fraction of cells in G2/M phase, but these effects were not seen in cells treated with tunicamycin. In line with these findings were the effects of RASSF1 inhibition in apoptosis. Tunicamycin exposure significantly induced TUNEL‐positivity in HEK293FT cells, but this effect was abolished when RASSF1 was inhibited (Fig. 7). Thus, RASSF1 is required for the effects of tunicamycin on cell apoptosis.

## Discussion

In the present study, we applied a novel *in silico* approach based on the analysis of RNA‐Seq data to identify UPR‐associated genes. Our analysis identified the tumor suppressor gene *RASSF1* that is involved in Ras signaling, as a UPR target gene. The premise of our analysis is that coordination analysis of gene expression can be applied to genetically diverse specimens and reveal regulatory relationships between genes. We tested our hypothesis by assessing transcripts of which the transcriptome exhibits co‐regulation with the transcriptome of UPR target genes in outbred deer mouse specimens. Our analyses indicated that *RASSF1* is highly co‐expressed with the major UPR chaperone *BiP/GRP78*. Furthermore, this co‐regulation is extended beyond the individual levels of expression, to the whole transcriptome, when the correlation of each and every gene was evaluated in comparison, between *RASSF1* and *BiP/GRP78*. This association was readily detectable when additional UPR genes were interrogated, and was present in both deer mouse fibroblasts and human liver specimens, albeit the fact that in the former was more pronounced, likely because it was assessed in cells as opposed to whole tissue samples. The only exception was recorded with *DDIT3/CHOP* that exhibited more relaxed coordination at the transcriptome level with *RASSF1* and is consistent with the fact that *DDIT3/CHOP* is also regulated by alternative to UPR pathways.

Strong evidence regarding the functional integration of RASSF1 to UPR signaling was obtained after subjecting the *RASSF1*‐correlated transcriptome to GO analyses, which showed high enrichment for ER stress‐associated biological processes. Among those, involvement with UPR‐associated cell death was predicted, especially in relation to PERK signaling. *In silico* analysis for transcription‐binding sites to *RASSF1* promoter pointed to the presence of ATF4‐binding sites, which is an established transducer of PERK signaling. These predictions were all subsequently confirmed by a combination of promoter‐reporter assays and chromatin IP studies that indeed demonstrated that ATF4 activates and physically interacts with the *RASSF1* promoter. Functional studies regarding the implications of RASSF1 into UPR signaling suggested that RASSF1 is required for cell cycle arrest and ER stress‐induced apoptosis, in response to tunicamycin exposure.

RASSF1 is an established tumor suppressor that induces cell cycle regulation and apoptosis and is inactivated in various cancers by hypermethylation or mutations. Nevertheless, no connection with the UPR was established for RASSF1 before. The present findings suggest that ATF4 activation downstream of PERK during ER stress activates, at the transcriptional level, RASSF1, which in turn induces cell cycle arrest and stimulates apoptosis. The proposed integration of RASSF1 into UPR signaling suggests that *RASSF1* activation may contribute to UPR‐associated pathologies at which excessive cell death is recorded. Conversely, in the context of anticancer therapy, these findings imply that UPR activation may be beneficial in cancers that are RASSF1‐dependent. Furthermore, DAXX was recently found as a new type of protein‐folding enabler [40], and it also plays a critical role in the p53‐mediated *RASSF1A* inactivation [41]. And RASSF1A associates with DAXX and MDM2 in the nucleus, promoting MDM2 self‐ubiquitination by the disruption of MDM2‐DAXX‐HAUSP complex [42]. These results may indicate the involvement of RASSF1 in the protein‐folding network.

RASSF1A and RASSF1C are two well‐studied RASSF1 isoforms. RASSF1A reduces cell proliferation and stimulates apoptosis while RASSF1C functions as an oncogene and shows the opposite activities [43, 44]. The remarkably higher *RASSF1A* induction by tunicamycin in HEK293FT cells, in combination with the abrogation of tunicamycin‐induced apoptosis in cells subjected to shRNA‐mediated RASSF1 inhibition, suggests that it is RASSF1A that mainly mediates apoptosis when UPR is induced. That during RASSF1 knockdown, G2/M arrest was only induced in the absence of tunicamycin, is likely indicative of the fact that during ER stress, arrested cells have already been sensitized towards apoptosis. Therefore, no considerable changes are recorded in their G2/M fraction during stress, nevertheless, apoptosis was significantly alleviated during tunicamycin treatment, when RASSF1 expression was compromised. BH3‐only sensor *BBC3/PUMA*, a PERK/eIF2α dependent pro‐apoptotic gene [27, 28] can be activated by RASSF1A [17, 38] and plays an important role in the ER stress‐induced apoptosis [45]. However, it is worth noting that other BH3‐only proteins, such as Bid and Bim can activate apoptotic signaling independent of BBC3/PUMA during ER stress [33, 46, 47].

Besides the significance of attributing RASSF1 UPR‐associated functionality, the present study illustrates how coordination analysis of gene expression may reveal causative associations between genes and biochemical pathways. In addition, GO analyses based on the enrichment of co‐regulated, as opposed to differentially expressed genes, may predict with high accuracy biological functions that can be validated experimentally. This highly versatile strategy is particularly applicable to the analysis of transcriptomic data from genetically diverse specimens, such as human samples, at which the observed variation in gene expression levels limits the statistical significance of conventional differential expression analyses and restricts their informative value. By focusing on the degree of transcriptomic coordination, as opposed to the magnitude of differential expression, it is plausible to unveil associations that would remain unnoticed by conventional approaches.

## Materials and methods

### *In silico* analysis of RASSF1 transcript

The RNA‐Seq data used here have been published [8, 48] and deposited in GEO (Accession numbers: GSE129534 and GSE130970). The flowchart of the process and analysis were described previously [8]. Briefly, Pearson's correlation coefficients were calculated between the whole transcriptome as obtained by the RNA‐Seq analysis and the transcripts indicated. Subsequently, the coordination between the UPR‐associated transcripts and *RASSF1* was calculated as the correlation of their Pearson's *R* values. For the Gene Ontology Enrichment analysis, the transcripts were sorted according to the *R* values of the whole transcriptome versus *RASSF1* and the identification of associated biological processes was performed using the gene ontology online platform [49, 50] at which the list of genes exhibiting *P* < 0.05 (Pearson's). The putative transcription factor‐binding sites of *RASSF1* promoter were analyzed using matinspector [51].

### Cell culture

HFFF2 (Sigma‐Aldrich, St. Louis, MO, USA) and HEK293FT (Invitrogen, Carlsbad, CA, USA) cells were cultured in the Dulbecco's Modified Eagle's Medium (DMEM; Corning, Glendale, AZ, USA) supplemented with 10% fetal bovine serum (Gibco, Grand Island, NY, USA), 100 U·mL^−1^ penicillin, 100 μg·mL^−1^ streptomycin, and 0.292 mg·mL^−1^ l‐glutamine (HyClone, Marlborough, MA, USA). Cells were maintained at 37 °C in a humidified environment with 5% CO_2_ and 95% air. For ER stress induction, cells were split into six‐well plates, at 300 000 cells/well, and cultured for 24 h. Then, cells were treated with either tunicamycin (5 μg·mL^−1^; Sigma) or thapsigargin (3 μm; Tocris, Minneapolis, MN, USA) with or without the addition of ISRIB (500 nm; Sigma) for 5 h, immediately followed by RNA extraction.

### RASSF1 luciferase reporter constructs

The genomic DNA was extracted from HFFF2 cells using DNeasy Blood & Tissue Kit (Qiagen, Germantown, MD, USA) according to the supplied protocol. The *RASSF1* promoter region (−930 to +38 relative to the transcription initiation site) was amplified by PCR using 100 ng genomic DNA, Q5 High‐Fidelity DNA polymerase (New England BioLabs, Ipswich, MA, USA), and the primers 1 (forward) (5′‐GCTGGAGCGAGAAAACAGAG) and 2 (reverse) (5′‐CAATGGAAACCTGGGTGCAG). The PCR product size was 969 base pairs. Following PCR, the generated fragment was subcloned into a pCR‐Blunt II‐TOPO vector (Invitrogen, Carlsbad, CA, USA). Then, the target fragment, co‐digested by KpnI and EcoRV (New England BioLabs), was subcloned into the KpnI and EcoRV sites of pBV‐Luc vector [52] (a gift from Bert Vogelstein; Addgene plasmid # 16539; http://n2t.net/addgene:16539; RRID: Addgene_16539), carrying a firefly luciferase coding sequence under the control of a minimal promoter. All constructs were confirmed by sequencing.

### Luciferase assay

HEK293FT cells were co‐transfected with the RASSF1 luciferase reporter plasmid, and pRK‐ATF4 expression plasmid [53] (a gift from Yihong Ye; Addgene plasmid # 26114; http://n2t.net/addgene:26114; RRID: Addgene_26114) or pRK‐ATF4 ΔC (1–275) expression plasmid [53] (a gift from Yihong Ye, Addgene plasmid # 26118; http://n2t.net/addgene:26118; RRID: Addgene_26118) using Lipofectamine 3000 transfection reagent (Invitrogen) according to the manufacturer's protocol. Luciferase activity in cell lysates was measured using luciferase assay system (Promega, Madison, MI, USA). Luciferase activity was normalized by the amount of the total protein.

### Chromatin immunoprecipitation assay

Chromatin immunoprecipitation (ChIP) assay was performed using the ChIP kit (Abcam, Waltham, MA, USA; ab500) according to the supplied protocol. Briefly, HEK293FT cells were exposed to 5 μg·mL^−1^ tunicamycin (Sigma‐Aldrich) for 5 h, cross‐linked with 1.1% formaldehyde (Thermo Scientific, Waltham, MA, USA; Cat# 28906) for 10 min at room temperature, and quenched in 0.125 m glycine. The cells were then incubated with lysis buffer and sonicated to produce 200–500 base pair DNA fragments. DNA fragments were immunoprecipitated from the cell lysates using anti‐ATF4 antibody (Abcam; ab184909) or rabbit IgG (Abcam; ab171870), and immunoprecipitates were recovered by the addition of DNA purifying slurry. After reverse crosslinking and washing, purified DNA was quantified by SYBR Green real‐time PCR (Bio‐Rad, Hercules, CA, USA) using specific primers (Table 2). The samples added rabbit IgG was used as a control. Data were expressed as the percentage of input.

**Table 2:** Oligonucleotide Primers for RT‐qPCR.

Name	Forward (5′–3′)	Reverse (5′–3′)	Product (bp)
*ATF4*	CCCCAGACGGTGAACCCAAT	CTGGAGTGGAGGACAGGACC	121
*BBC3*	ACGACCTCAACGCACAGTAC	CTGGGTAAGGGCAGGAGTC	112
*CCNA2*	AGCATGTCACCGTTCCTCCT	CCAGGGCATCTTCACGCTC	132
*GAPDH*	AGAAGGTGGTGAAGCAGGCG	AAGGTGGAGGAGTGGGTGTC	109
*RASSF1*	TGCCCAGATCAACAGCAACC	CTGCAAGGAGGGTGGCTTCT	130
*RASSF1A*	TTCACCTGCCACTACCGCTG	GTCTCCCACTCCACAGGCTC	122
*RASSF1C*	AATGACCTGGAGCAGCACGA	GTCTCCCACTCCACAGGCTC	103
*RASSF1* (ChIP)	GATCTCCCTCCTCCTCACCC	CCTGGTCCGGTTTGCTGAA	94

### Establishment of RASSF1 knockdown cells

The hRASSF1‐RNAi lentiviral vector pLV‐EGFP‐Puro shRNA and lentiviral‐carrying scrambled shRNA were constructed by VectorBuilder (Chicago, IL, USA). The RNAi target sequence against RASSF1 is AGACAGAAGTCTCCTCAATTT, and the scrambled shRNA was served as a control. The vector packaging and harvesting were performed by transfection of HEK293FT cells using PEI transfection reagent (Polysciences, Warrington, PA, USA). Briefly, HEK293FT cells were co‐transfected with 1.5 μg of pMD2.G, 4.5 μg of psPAX2, and 6 μg of RASSF1 or control shRNA and cultured for 48 h. The supernatant containing lentiviral vectors was collected and filtered and then mixed 1 : 1 volume with complete culture media and added to cells. About 8 μg·mL^−1^ of polybrene was also added to the virus to increase transduction efficiency. Cells were selected with 2 μg·mL^−1^ of puromycin and the knockdown efficiency was confirmed by western blot.

### Western blots

Whole‐cell lysates were obtained from RASSF1 and control shRNA transfected HEK293FT cells treated with tunicamycin (5 μg·mL^−1^) for 5 h. The cells were harvested with RIPA lysis buffer (Thermo Fisher, Waltham, MA, USA). Lysates were sonicated for 30 s, and the protein concentration was measured by DC protein assay (Bio‐Rad). Protein samples (30 μg each) were separated by 4–12% PAGE Gel (GenScript, Piscataway, NJ, USA) and then transferred onto PVDF membranes (Millipore‐Sigma, Burlington, MA, USA). Membranes were blocked with 5% nonfat milk for 60 min at room temperature and incubated overnight at 4 °C with recombinant anti‐RASSF1 rabbit monoclonal antibody (1 : 500, ab126764; Abcam) or anti‐α‐Tubulin monoclonal mouse antibody (1 : 5000, T9026; Sigma). After washing, membranes were incubated for 1 h at room temperature with horse radish peroxidase (HRP) conjugated goat anti‐rabbit IgG secondary antibody (1 : 10 000; Abcam) or goat anti‐mouse IgG secondary antibody (1 : 10 000; ThermoFisher) at room temperature. The immobilized proteins were detected using the enhanced chemiluminescence reagent plus (PerkinElmer, Waltham, MA, USA). Images were obtained with ChemiDoc™ Touch Imaging System (Bio‐Rad) and analyzed with image lab.

### RNA extraction, cDNA synthesis, and qPCR

RNA was extracted with a Qiagen RNeasy Plus Mini kit as per the manufacturer's recommendations (Qiagen). Complementary DNA (cDNA) synthesis was conducted using an iScript cDNA synthesis kit (Bio‐Rad) according to the supplied protocol. Quantitative PCR (qPCR) was performed on a T100 thermocycler (Bio‐Rad) using iTaq Universal SYBR Green Supermix (Bio‐Rad). Specific oligonucleotide primers for target gene sequences are listed in Table 2. Arbitrary units of target mRNA were normalized to the level of *GAPDH* expression.

### Cell cycle analysis

RASSF1 and control shRNA transfected HEK293FT cells were treated with tunicamycin (5 μg·mL^−1^) for 24 h and then fixed in 70% ethanol overnight at 4 °C. Cells were washed once with PBS and labeled with 1 μg·mL^−1^ 4′,6‐diamidino‐2‐phenylindole (DAPI) in PBS/0.1% Triton X‐100 solution for 30 min at room temperature. Cell cycle phases were analyzed with BD LSR II flow cytometer (BD Biosciences, Franklin, NJ, USA).

### Cell apoptosis assay

The apoptotic cells were detected using the *In Situ* Cell Death Detection Kit, Fluorescein (Roche, Indianapolis, IN, USA) according to the supplied protocol. Briefly, cells were treated with tunicamycin (5 μg·mL^−1^) for 24 h, then washed with PBS and fixed with freshly prepared 2% paraformaldehyde for 1 h at room temperature. The cells were permeabilized with 0.1% Triton X‐100 solution for 2 min on ice and then labeled with TUNEL reaction mixture for 1 h at 37 °C in a humidified atmosphere in the dark. The cells were resuspended in FBS and smeared over a coverslip. The number of apoptotic cells was counted with a fluorescence confocal microscope (Carl Zeiss LSM 700, Carl Zeiss AG, Oberkochen, Germany) and analyzed with imagej   (NIH, Bethesda, MD, USA).

### Statistical analysis

Statistical analyses were performed using prism software (version 9.2.0; GraphPad Software, San Diego, CA, USA). The data were expressed as mean ± SEM unless specified otherwise. Results were analyzed using an unpaired two‐tailed *t*‐test, one‐way ANOVA followed by the Tukey's multiple comparison test or Pearson's correlation as indicated. *P* < 0.05 was considered significant.

## Conflict of interest

The authors declare no conflict of interest.

## Author contributions

YZ and HK conceived and designed the project; YZ, K‐TH‐D, XD, VS, and CL acquired the data; YZ and HK analyzed and interpreted the data; HK and EB supervised the project and provided resources; HK and YZ wrote the original draft. All authors discussed the results and approved the final manuscript.

## Supporting information


**Fig. S1.** Whole transcriptome coordination analysis between *RASSF1* and each of *BBC3‐*, *GADD45A‐*, *RCAN1‐*, and *CCNA2*‐associated transcriptomes.

**Fig. S2.** RASSF1 knockdown by shRNA alters the cell population distribution in different stages of cell cycle under ER stress conditions.

**Table S1.** The calculation of Pearson's R values for whole transcriptome coordination of *RASSF1* and UPR target genes *HSPA5/BiP*, *DNAJB9*, *HSP90B1*, *ATF4*, *DNAJC3*, and *DDIT3* in primary fibroblasts of deer mice.

**Table S2.** The calculation of Pearson's R values for whole transcriptome coordination of *RASSF1* and UPR target genes *HSPA5/BiP*, *DNAJB9*, *HSP90B1*, *ATF4*, *DNAJC3*, and *DDIT3* in human liver specimens.

**Table S3.** Gene ontology enrichment analysis for transcripts that exhibited significantly (*P* < 0.05, Pearson's) positive, or positive and negative correlated expression to *RASSF1* in *Mus musculu*s or *Homo sapiens* genome.

## Data Availability

All the relevant data are within the manuscript and the [Link], [Link], [Link], [Link], [Link] file. The whole transcriptome coordination analysis data and the complete data set of gene ontology enrichment analysis are provided in Tables [Link], [Link], [Link].
