# Corrigendum to: RASSF1 Is identified by transcriptome coordination analysis as a target of ATF4. https://doi.org/10.1002/2211‐5463.13569

**DOI:** 10.1002/2211-5463.13801

**Published:** 2024-04-08

Within the Materials and Methods section of the paper by Zhang *et al*. [1], the wrong GEO accession numbers were provided. The correct GEO accession numbers are given below:

“The RNA‐Seq data used here have been published [8,48] and deposited in GEO (Accession numbers: GSE131429 and GSE130970).”

