# Supplementary material for: RASSF1 is identified by transcriptome coordination analysis as a target of ATF4
Source: FEBS Open Bio. 2023 Feb 14;13(3):556–69. doi: 10.1002/2211-5463.13569 (PMC9989924; doi:10.1002/2211-5463.13569)
Supplement: Supplementary file 1 — Fig. S1. Whole transcriptome coordination analysis between RASSF1 and each of BBC3‐, GADD45A‐, RCAN1‐, and CCNA2‐associated transcriptomes. [file FEB4-13-556-s001.pdf]

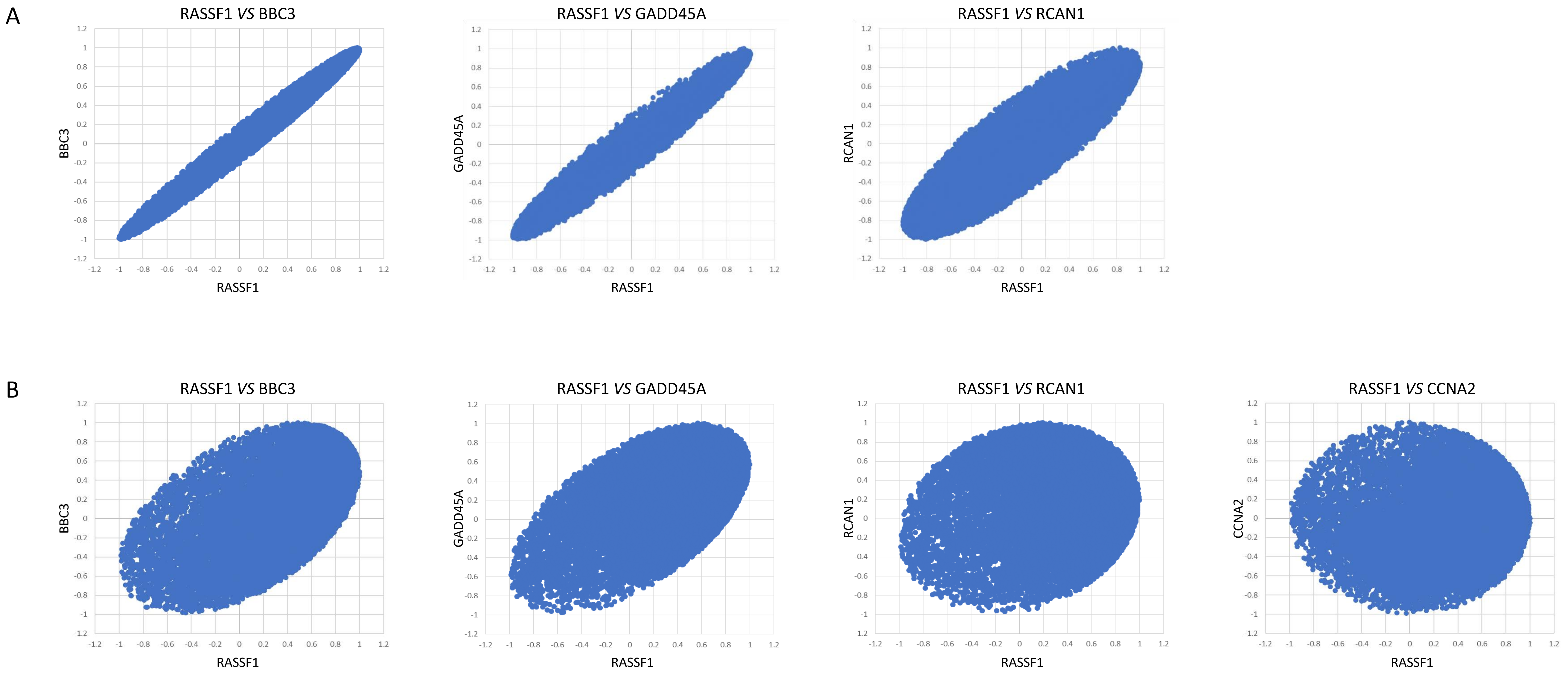

Fig. S1. Whole transcriptome coordination analysis between *RASSF1* and each of *BBC3, GADD45A, RCAN1* and *CCNA2*-associated transcriptomes. The transcriptome of *RASSF1* showed tight association with those of *BBC3* and *GADD45A*, two PERK-eIF2α dependent pro-apoptotic genes, but the correlation was reduced with the transcriptome of *RCAN1*, an ATF6-dependent pro-survival regulator. In line with the RNA expression data of Fig. 3, the *RASSF1*-associated transcriptome was coordinated with the *BBC3*-associated transcriptome but not with the *CCNA2*-associated transcriptome. Data were obtained from deer mouse fibroblasts RNA-Seq data for *BBC3*, *GADD45A* and *RCAN1* (8), and human liver RNA-Seq data for *BBC3*, *GADD45A, RCAN1* and *CCNA2* (48) (*CCNA2* was not detected in the deer mouse data).
