# Supplementary material for: RASSF1 is identified by transcriptome coordination analysis as a target of ATF4
Source: FEBS Open Bio. 2023 Feb 14;13(3):556–69. doi: 10.1002/2211-5463.13569 (PMC9989924; doi:10.1002/2211-5463.13569)
Supplement: Supplementary file 2 — Fig. S2. RASSF1 knockdown by shRNA alters the cell population distribution in different stages of cell cycle under ER stress conditions. [file FEB4-13-556-s004.pdf]

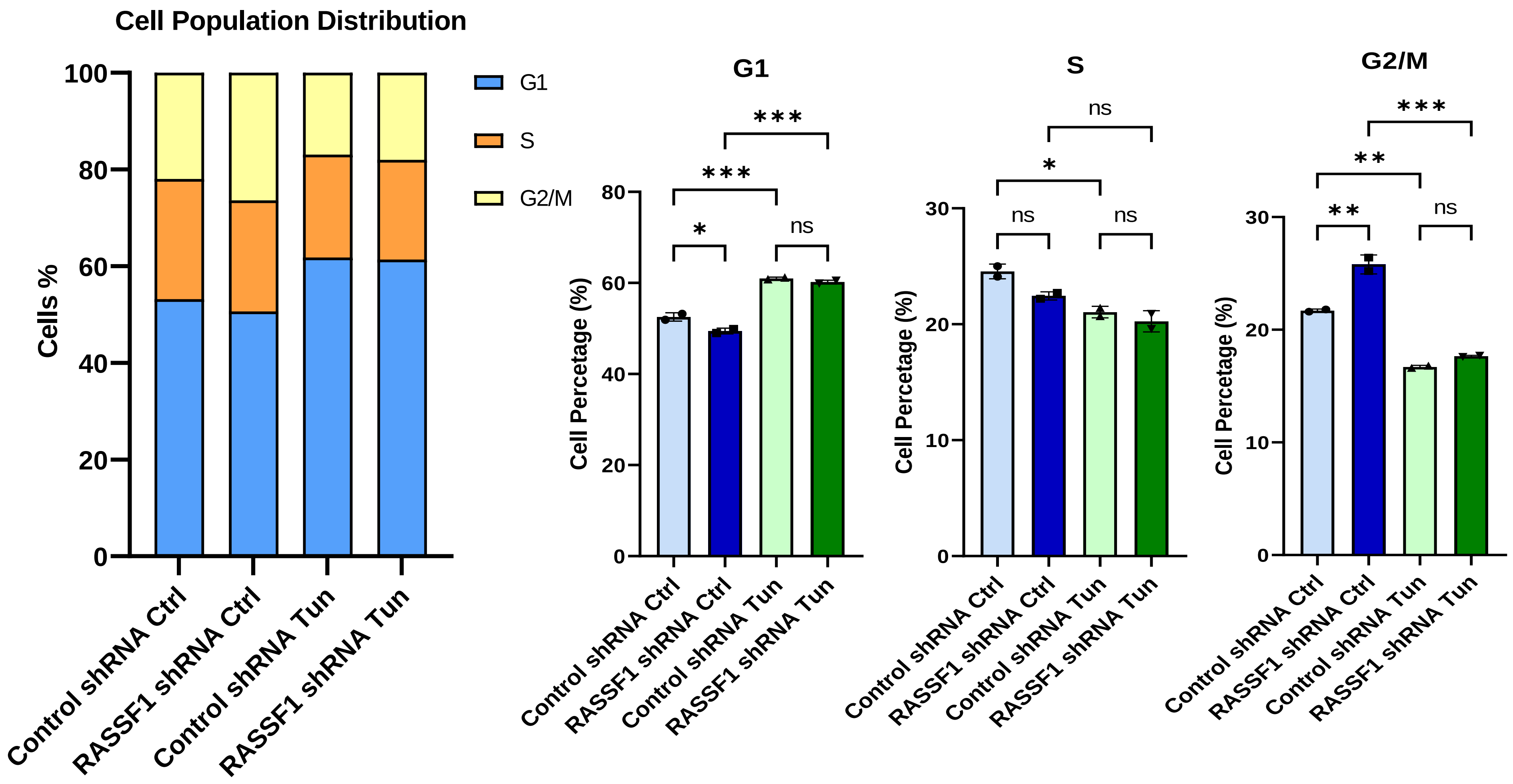

Fig. S2. RASSF1 knockdown by shRNA alters the cell population distribution in different stages of cell cycle under ER stress conditions. HEK293FT cells were transfected with hRASSF1- or control shRNA and treated with tunicamycin (5 µg/mL). The cell populations in different stages of cell cycle were measured with flow cytometry (n = 2 biological replicates). Control shRNA Ctrl – cells transfected with scrambled shRNA and without tunicamycin treatment, RASSF1 shRNA Ctrl – cells transfected with hRASSF1-shRNA and without tunicamycin treatment, Control shRNA Tun – cells transfected with scrambled shRNA and treated with tunicamycin, RASSF1 shRNA Tun – cells transfected with hRASSF1-shRNA and treated with tunicamycin. P values were calculated with one-way ANOVA followed by Tukey's multiple comparisons test. Data represent the mean ± SEM. * $P < 0.05$, ** $P < 0.01$, *** $P < 0.001$, ns non-significant.
